# Comparison of the Self-Expandable Intra-Annular Navitor Prosthesis with the Balloon-Expandable, Intra-Annular Sapien 3 Prosthesis: A Propensity-Matched Analysis

**DOI:** 10.3390/jcm15124528

**Published:** 2026-06-11

**Authors:** Nazan Puluca, Melchior Burri, Julia Schreyer, Magdalena Erlebach, Felix Wirth, Caterina Campanella, Stephanie Voss, Markus Krane, Hendrik Ruge

**Affiliations:** 1 Department of Cardiovascular Surgery, Institute Insure, German Heart Center Munich, School of Medicine & Health, Technical University of Munich, Lazarettstrasse 36, 80636 Munich, Germany; puluca@dhm.mhn.de (N.P.); melchiorburri@msn.de (M.B.); schreyer@dhm.mhn.de (J.S.); erlebach@dhm.mhn.de (M.E.); wirth@dhm.mhn.de (F.W.); campanella@dhm.mhn.de (C.C.); vosss@dhm.mhn.de (S.V.); 2 DZHK (German Center for Cardiovascular Research)—Partner Site Munich Heart Alliance, 80636 Munich, Germany

**Keywords:** transcatheter aortic valve implantation, TAVR, Sapien, Navitor, self-expandable, balloon-expandable

## Abstract

**Background:** The study aims to compare the short-term clinical outcomes of transcatheter aortic valve implantation (TAVI) with the novel self-expandable, intra-annular Navitor valve (NAV) and the balloon-expandable, intra-annular Sapien 3 Ultra valve (S3U). **Methods:** From a single-center TAVI database, patients receiving NAV and S3U were identified. We applied 1:2 propensity score matching for the selected variables (gender, age, aortic valve perimeter, area, diameter, mean aortic valve gradient, EuroScore2, coronary artery disease (CAD), previous stroke and previous pacemaker implantation), resulting in 153 patients. **Results**: Clinical outcomes at 30 days of 51 patients with NAV [mean age: 80.4 ± 6.7 years; 51% female; mean annulus diameter: 24.1 ± 1.40 mm; EuroScore2: 3.4 ± 3.1%] and 102 patients with S3U [mean age: 79.9 ± 6.5 years (*p* = 0.7); 51% female (*p* > 0.99); mean annulus diameter: 24.1 ± 1.4 mm (*p* > 0.99); EuroScore2: 3.2 ± 2.7 (*p* = 0.7)] were analyzed according to VARC-3 recommendations. Post-TAVI aortic valve mean (S3U: 11.0 [3–27] mmHg; NAV: 7 [3–15] mmHg; *p* < 0.001) and maximum (S3U: 22 [6–44] mmHg; NAV: 12 [5–28] mmHg; *p* < 0.001) gradients at discharge were significantly lower with NAV, whereas the effective orifice area (EOA) of the aortic valve measured significantly larger with NAV (S3U: 1.5 [0.8–3.8] cm^2^; NAV: 2.1 [0.9–3.5] cm^2^; *p* < 0.001). Rates of no to mild paravalvular regurgitation (PVL) were 92.1% after NAV and 91.2% after S3U implantation (*p* = 0.15), mild to moderate PVL were 2.0% after NAV vs. 2.9% after S3U (*p* = 0.1) and moderate PVL were 2% after NAV and 1% after S3U (*p* = 0.07). None of the patients had a severe regurgitation. Severe patient–prosthesis mismatch (PPM) occurred significantly less with NAV (S3U: 14.7%; NAV: 7.8%; *p* = 0.002). One (1%) non-disabling stroke occurred within the S3U group and none occurred within the NAV group (*p* = 0.1). Life-threatening (S3U: 2.9%; NAV= 1%; *p* > 0.99) and major (S3U: n = 2.9; NAV: 0%; *p* = 0.55) bleeding events were comparable between both groups. The incidence of major (S3U: 2.9%; NAV: 2.0%; *p* > 0.99) vascular complications and the need for permanent pacemaker implantation (S3U: 9.8%; NAV: 11.8%; *p* = 0.8) were comparable in both groups. The 30-day mortality rate was 0.7% [1 in NAV group (2%), none in S3U; *p* = 0.3]. **Conclusions:** In conclusion, at 30-day follow-up, the self-expanding intra-annular Navitor valve demonstrated excellent acute safety and superior early hemodynamic performance, characterized by significantly lower transvalvular gradients and lower rates of severe PPM compared to the balloon-expandable Sapien 3 Ultra. However, whether these acute hemodynamic advantages translate into superior long-term clinical outcomes remains to be determined in long-term follow-up studies.

## 1. Introduction

Transcatheter aortic valve implantation (TAVR) has emerged as the predominant treatment option for aortic valve stenosis beyond high-risk patients in the Western world [1]. Following two randomized controlled trials comparing surgical aortic valve replacement (SAVR) and TAVR in low-surgical-risk cohorts [2,3,4], transfemoral TAVR has become a class I indication for low-risk patients with symptomatic, severe aortic valve stenosis in the current AHA guidelines on the management of valvular heart disease [5]. Therefore, cardiac surgeons and cardiologists face challenges regarding patients’ lifetime management, as “healthier” patients may require more than one aortic valve replacement. In addition to hemodynamic performance, long-term valve durability and future coronary access become more important for these individuals.

Current modifications of transcatheter heart valves (THVs) aim to improve valve performance, focusing on lifetime management needs. The Navitor valve is a second-generation valve following its predecessor, the Portico valve [6]. For the novel self-expandable Abbott Navitor THV, an intra-annular leaflet position and its large stent cells provide unhampered coronary access and low transvalvular gradients. A modified outer skirt aims to improve paravalvular sealing and achieve a reduction in paravalvular regurgitation [7,8]. In the current generation of the most widely used balloon-expandable Sapien 3 Ultra Resilia THV, pericardial tissue processing aims to improve valve durability [9]. In addition, an outer skirt for improved PVL sealing has been added to the 29 mm valve size [10].

Here, we aimed to compare early clinical outcomes of the Abbott Navitor (Abbott Medical, Ort, USA) valve with the Edwards Sapien 3 Ultra (Edwards Lifesciences, Irvine, USA) in a propensity-matched analysis. Recent comparative data between contemporary intra-annular transcatheter heart valves remain limited. However, emerging evidence from observational head-to-head analyses suggests potential differences in clinical outcomes between balloon-expandable and novel self-expanding platforms, particularly regarding stroke incidence, pacemaker implantation, and paravalvular leaks. These findings highlight the need for further comparative analyses in real-world cohorts.

## 2. Materials and Methods

**Figure 1:**
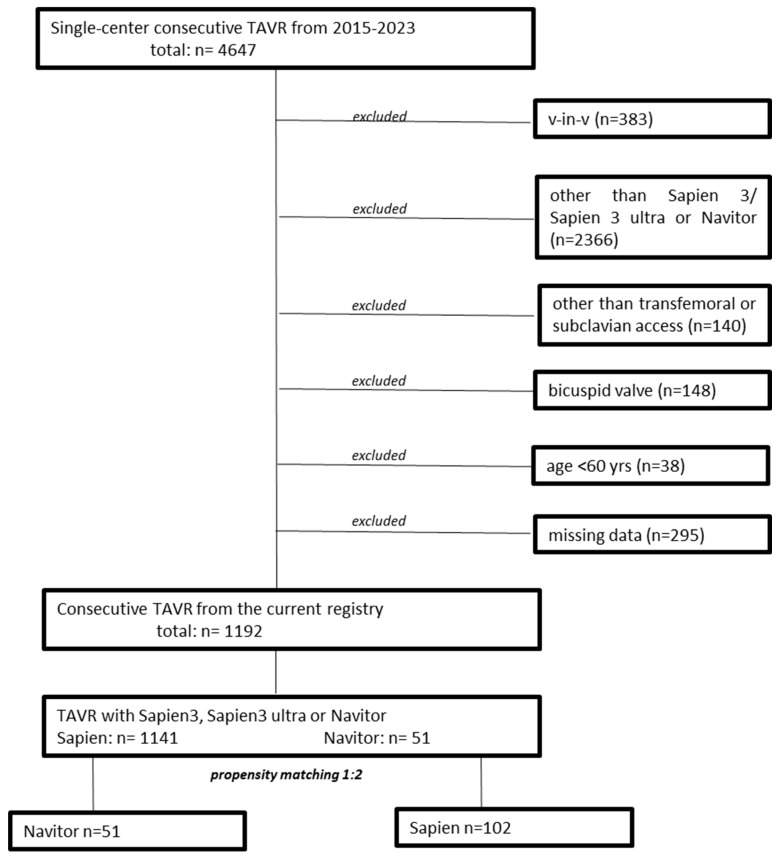
Flow chart.

A total of 4647 patients who underwent transcatheter aortic valve replacement (TAVR) at the German Heart Center Munich between 2015 and 2023 were included from a single-center database. Patients treated from 2015 onward were selected, as the Edwards SAPIEN 3 (Irvine, CA, USA) valve received CE Mark approval shortly before. Only patients undergoing subclavian or transfemoral access TAVI were included. Further exclusion criteria are listed in Figure 1. A 1:2 propensity-score-matching ratio was chosen to optimize statistical power and minimize estimate variance, given the large imbalance between the available Sapien 3 Ultra control pool and the novel Navitor (Abbott, St. Paul, MN, USA), cohort. Propensity score matching (1:2) was preferred over Inverse Probability of Treatment Weighting (IPTW) to avoid the severe variance inflation and statistical instability often caused by extreme weights in highly unbalanced cohorts. Notably, the inclusion of the EuroSCORE II in the matching model served to adjust for the chronological shift in patient risk profiles and baseline clinical characteristics that occurred over the multi-year enrollment period (2015–2023). While individual parameters such as specific calcium scores, left ventricular function, and frailty indices were not entered as separate covariates, they were implicitly adjusted for via the strict matching of the baseline mean aortic gradient, detailed annular CT metrics (area, perimeter, diameter), and the comprehensive EuroSCORE II, which directly incorporates ventricular function and clinical risk surrogates. Furthermore, the cohort was highly homogeneous regarding procedural approach, as all matched patients presented with tricuspid aortic valve anatomy and underwent subclavian or transfemoral access TAVI.

The choice of the implanted prosthesis platform was left to operator preference. During the inclusion period, this preference was primarily determined by device availability, operator training schedules, and the institutional initiative to integrate the novel Navitor platform into clinical routine. No systematic anatomical or clinical pre-selection protocols were applied, provided the patient’s aortic root morphology met the standard instructions for use for either device.

Clinical data were analyzed for hemodynamic outcomes and Valve Academic Research Consortium-3 (VARC-3)-defined composite endpoints.

### Statistical Analysis

**Figure 2:**
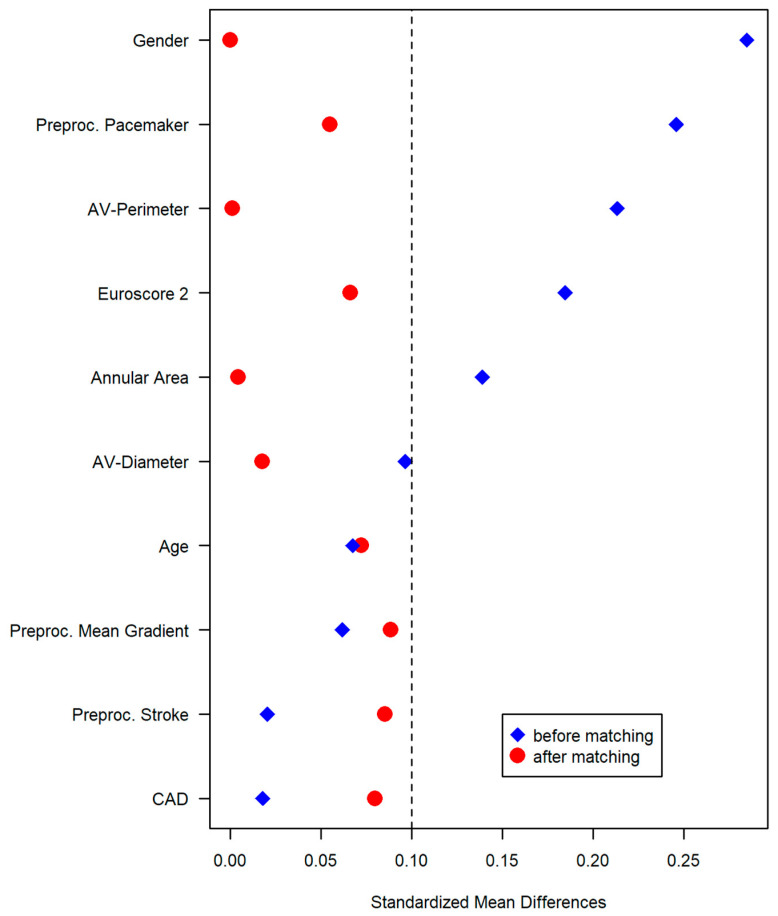
Loveplot—standardized mean difference before and after matching.

Propensity score matching was performed including 10 variables: gender, age, aortic valve perimeter, area, diameter, mean aortic valve gradient, EuroScore2, coronary artery disease (CAD), previous stroke and previous pacemaker implantation. The distribution of included variables before and after matching is shown in Figure 2.

Statistical data are reported as follows:

Means + SD were reported for normally distributed and medians (range) for non-normally distributed values. Categorical variables are expressed as absolute numbers and percentages. Comparative analysis was performed using Student’s t-test or Fisher’s exact test.

A value of <0.05 was considered to indicate statistical significance.

Statistical analysis was performed using R (Version 4.4.3).

## 3. Results

### 3.1. Baseline Characteristics

Table 1 presents the baseline characteristics of all patients before and after matching. There were no statistically significant differences between the Navitor and ES3 groups regarding demographics, risk scores or aortic anulus dimensions. Aortic annulus area measured 447 ± 51 mm in the Navitor group vs. 448.3 ± 50.5 mm in the ES3 group (*p* > 0.99). There was no statistically significant difference in aortic annulus perimeter after matching (Navitor group: 7.6 ± 0.4 cm; ES3 group: 7.7 ± 0.4 cm; *p* > 0.99).

### 3.2. Procedural Details

Within the Navitor group, valves of sizes 27 and 29 were mainly implanted, whereas in the ES3 group, sizes 23 and 26 were used in the majority of patients (Table 2).

Regarding the technical aspects, there was a significant difference between both groups in the amount of contrast medium used. Fluoroscopy time was higher within the Navitor group (S3U: 10.3 [2.8–35.4] minutes; NAV: 12.2 [5.9–31.8] minutes; *p* = 0.011).

Higher rates of pre-and postdilatation were observed in the Navitor group in comparison to the ES3 group (predilatation: S3U: n = 18 [17.6%]; NAV: n = 50 [98%]; *p* < 0.001; postdilatation: S3U: n = 29 [28.4%]; NAV: n = 23 [45.1%] minutes; *p* = 0.047).

### 3.3. Hemodynamic Performance

**Figure 3:**
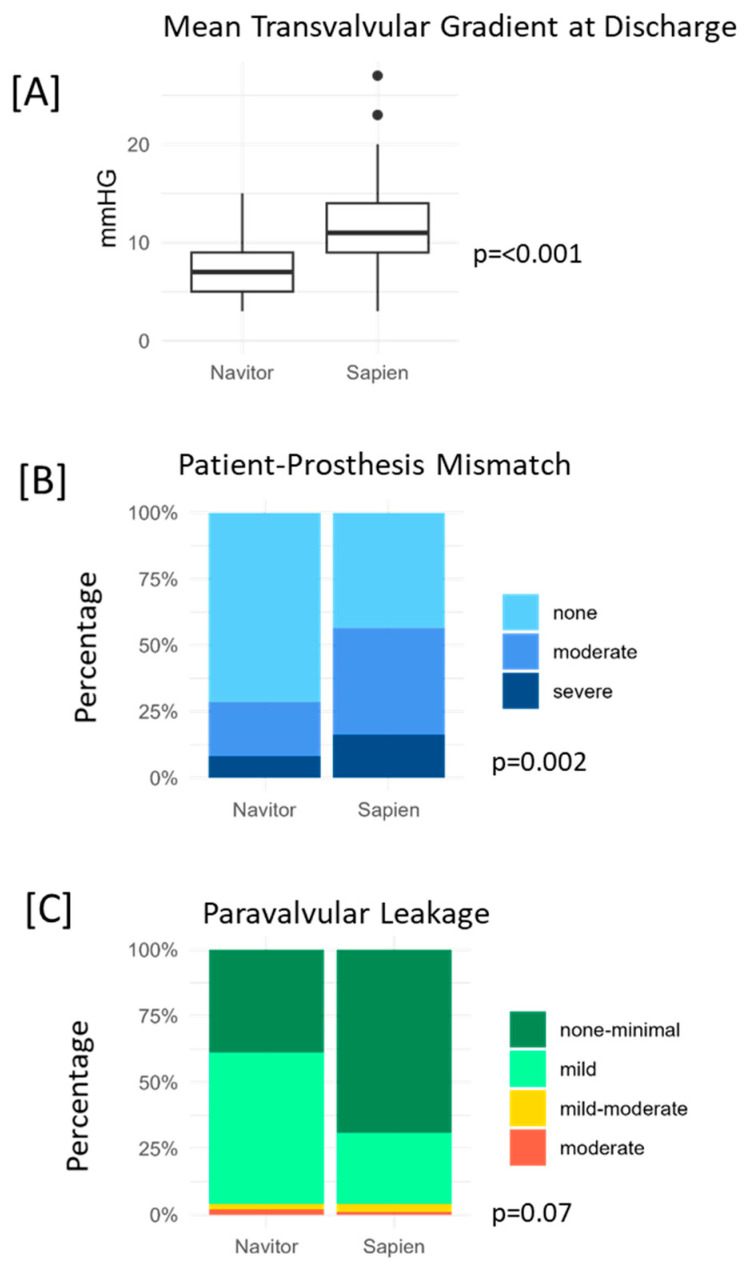
Hemodynamic results. (**A**) The horizontal centerline inside each box represents the median, while the lower and upper boundaries represent the first (Q1) and third (Q3) quartiles, respectively. (**B**,**C**) Data labels and bars represent percentages (%) within each respective valve group.

Post-TAVI aortic valve mean (S3U: 11.0 [3–27] mmHg; NAV: 7 [3–15] mmHg; *p* < 0.001) and maximum (S3U: 22 [6–44] mmHg; NAV: 12 [5–28] mmHg; *p* < 0.001) gradients at discharge were significantly lower with NAV (Figure 3A).

EOA was significantly larger in Navitor patients (S3U: 1.5 [0.8–3.8] cm^2^; NAV: 2.1 [0.9–3.5] cm^2^; *p* < 0.001).

Severe patient–prosthesis mismatch occurred significantly less frequently (Figure 3A) in patients receiving a Navitor valve (S3U: 14.7%; NAV: 7.8%; *p* = 0.002).

The rates of no to mild paravalvular regurgitation were 92.1% after NAV implantation and 91.2% after S3U (*p* = 0.15), and rates of mild to moderate PVL were 1.96% after NAV vs. 2.9% after S3U (*p* = 0.1). Moderate PVL occurred in 2% of patients after Navitor implantation and 1% after S3U implantation (*p* = 0.07). None of the patients had a severe regurgitation (Figure 3C).

### 3.4. In-Hospital Outcomes

One (1%) non-disabling stroke occurred within the S3U group compared to none within the NAV group (*p* = 0.1). Life-threatening (S3U: 2.9%; NAV n = 1%; *p* > 0.99) and major (S3U: n = 2.9; NAV: 0%; *p* = 0.55) bleeding events were comparable between both groups. The incidence of major (S3U: 2.9%; NAV: 2.0%; *p* > 0.99) vascular complications and the need for permanent pacemaker implantation (S3U: 9.8%, NAV: 11.8%; *p* = 0.8) were comparable in both groups. Thirty-day mortality was 0.7% [one in NAV group (2%), none in S3U, *p* = 0.3; Table 3].

Conversion to surgical aortic valve replacement was required in two Navitor patients due to acute valve migration. In both cases, the patients remained hemodynamically stable during the event, and the valve migrated aortically. These two events occurred early during our center’s initial learning curve with the platform, at a time when the specific release mechanics were not yet fully appreciated—specifically, that the final detachment of the valve frame from the delivery system requires sufficient time and distinct manipulation to completely disengage the radiopaque hooks at the crown. Both patients underwent successful emergency conversion to surgical aortic valve replacement via median sternotomy and survived the acute postoperative course without further complications. None of the patients within the Sapien group required surgery conversion (*p* = 0.11).

### 3.5. VARC-3-Defined Composite Outcomes

VARC-3-defined technical success rate was 94.1% for the Navitor group and 93.1% (*p* > 0.99) for the Sapien group (Table 4).

Regarding device success, the rate was 92.2% for patients undergoing Navitor implantation and 81.4% (0.8) for patients who received an S3U.

## 4. Discussion

### 4.1. Summary of Key Findings

Recently, emerging comparative data evaluating the Navitor valve against the SAPIEN 3 Ultra RESILIA platform have suggested potential differences in clinical outcomes, with lower rates of stroke, permanent pacemaker implantation, and paravalvular leak reported for the balloon-expandable platform. These findings contrast with the comparable safety outcomes observed in our cohort and underscore the heterogeneity of currently available data.

The main findings of our study can be summarized as follows:

1. The VARC-3-defined technical success rate and device success rate were comparable between both THV platforms, with a numerically higher device success rate for the Navitor THV.
2. The Navitor valve demonstrated superior early hemodynamic outcomes in terms of lower transvalvular gradients and a lower patient–prosthesis mismatch rate.
3. There was no significant difference in the occurrence of paravalvular leakage between both groups.
4. The pacemaker rate was similar with no statistical difference between patients who received a Navitor or Sapien 3 valve.
5. Life-threatening bleeding and major vascular complications were comparable for both groups.
6. A disabling stroke did not occur in any of the groups during the short-term follow-up.
7. There was no statistically significant difference regarding early mortality between NAV and S3U patients.

### 4.2. Technical and Device Success

Considering technical success, both valve types provided high safety outcomes for patients undergoing TAVR. Eckel et al. reported similar outcomes for the Navitor prosthesis in comparison to its predecessor, the Portico valve [11].

In comparison, Pelligrini et al. described a VARC-3-defined device success rate of 83.7% and a high technical success rate of 95.5% for the well-established Sapien Ultra platform [12].

Device success was higher for patients undergoing TAVR with the Navitor valve, which is mainly affected by the impact of superior hemodynamic outcomes. The OCEAN TAVI registry, a prospective study conducted in Japan, compared outcomes of transcatheter aortic valve implantation (TAVI) using various valve technologies in 1546 patients with aortic stenosis.

In the OCEAN-TAVI registry, 463 patients underwent TAVI using the Navitor™ valve, with a high procedural success rate of 97.5%. All patients who received the Navitor™ valve did not require conversion to surgical aortic valve replacement (SAVR) and there was no procedural mortality. The major vascular complication rate was low, at 0.8%, which is comparable to other contemporary TAVI systems.

### 4.3. Superior Hemodynamic Performance and PPM

One of the most important findings of this study was the superior early hemodynamic profile of the Navitor valve. Compared to the Sapien 3 platform, the Navitor valve demonstrated significantly lower mean transvalvular gradients. The landmark SMART trial previously demonstrated higher transvalvular gradients for the Sapien 3 platform in comparison to the self-expanding Evolut prosthesis in patients with a small aortic annulus, resulting in a markedly higher PPM rate. Those findings have been further corroborated by several other studies comparing self-expanding to balloon-expandable valves, such as the OPERA-TAVI registry, TAVI SMALL2, and the OCEAN TAVI registry [13,14,15].

Transvalvular gradients are inherently influenced by both left ventricular outflow tract characteristics and systemic hemodynamics. Specifically, transaortic valve gradients are dependent on transaortic flow, which is modulated by several left ventricular parameters (including stroke volume and left ventricular mass index) as well as prosthesis performance [16]. Because greater flow resulting from an increased cardiac output may artificially elevate gradients, a flow-adjusted gradient analysis could reduce baseline confounding regarding long-term outcome predictions. Furthermore, fluid dynamics—particularly turbulent versus laminar flow patterns—are heavily impacted by the underlying valve design. Varying lengths of valve frames can alter the velocity–time integral, potentially leading to smaller calculated effective orifice areas (EOAs) when utilizing the continuity equation. Therefore, accurate assessment of the EOA in echocardiographic analyses, achieved through proper Doppler alignment, remains key to preventing mathematical miscalculations, as the EOA is critical in determining whether patient–prosthesis mismatch exists.

Concurrently, the rate of patient–prosthesis mismatch was lower in the Navitor group (Figure 3B). PPM is a well-recognized and intensively discussed phenomenon associated with adverse outcomes, particularly in patients with smaller annular sizes [17]. While the long-term clinical impact of mild-to-moderate PPM remains a topic of active debate, studies have consistently shown independent associations with increased rates of heart failure hospitalization, structural valve degeneration, and overall mortality in cases of severe PPM [18,19,20]. That said, large-scale registries such as the TVT registry (Eng et al.) and clinical trials like PARTNER 3 have reported no significant survival differences related to PPM in certain subgroups, particularly those receiving small Sapien valves [2,21]. Eng et al. provided data from the TVT registry comparing 8100 propensity-matched pairs, showing no differences in 3-year mortality for patients with a small Sapien 3 (20 mm) in comparison to patients with larger valves (≥23 mm). Their data suggested that low baseline LVEF was a stronger predictor of increased mortality rather than severe PPM. In addition, insights from the PARTNER 3 trial did not demonstrate a survival difference at 5 years according to the extent of PPM when comparing 476 patients with small annuli to 879 with large annuli using the Sapien 3 platform [22].

Comprehensive evaluations involving noninvasive and invasive gradients frequently show disparities, which are not uncommon in clinical practice [23,24]. These discrepancies between echocardiographic and invasive measurements underscore the need for more nuanced evaluation methods, including flow-adjusted gradient analyses and multimodal evaluation approaches, to provide a complete picture of valve performance.

We acknowledge that the present analysis is limited to 30-day clinical and echocardiographic outcomes. However, a wealth of clinical evidence confirms that acute hemodynamic performance at discharge serves as a critical surrogate and a strong predictor of long-term valve durability and patient survival. Large-scale trials and registries have consistently demonstrated that elevated post-procedural gradients and the presence of severe prosthesis–patient mismatch (PPM) detected within the first 30 days are independently associated with accelerated structural valve degeneration (SVD), increased rates of heart failure hospitalization, and higher long-term mortality [13,14]. Furthermore, according to the Valve Academic Research Consortium (VARC-3) consensus, early hemodynamic assessment forms the mandatory baseline for defining late valve failure [15]. Consequently, the significantly lower mean transvalvular gradients and reduced incidence of severe PPM observed in the Navitor group in our study are not merely transient acute findings, but may represent a favorable hemodynamic profile potentially associated with improved long-term outcomes.

### 4.4. Paravalvular Leakage

Historically, self-expanding valves have been associated with a higher incidence of PVL compared to balloon-expandable valves. This has been a major concern, as moderate or severe PVL has consistently been linked to impaired long-term outcomes and increased mortality after TAVI. However, recent design improvements and modified implantation techniques, including optimized postdilatation balloon sizing strategies and cusp-overlay implantation techniques, have substantially mitigated this issue.

The international multicenter PORTICO NG study revealed that 2 out of 260 patients required a vascular plug during the index procedure because of moderate PVL in cases of very deep valve implantation. All remaining patients demonstrated no to mild PVL at 30-day follow-up [16,17]. While only short-term follow-up data are currently available, these findings may still be clinically relevant given the established association between early moderate-to-severe PVL and adverse long-term outcomes after TAVI [18].

In our study, PVL rates did not differ significantly between the Navitor and Sapien 3 groups. Notably, the Navitor’s adaptive sealing skirt appears to have effectively minimized PVL, consistent with findings from Eckel et al. These results suggest that the Navitor valve has addressed one of the most prominent limitations of earlier self-expanding platforms such as Portico, which demonstrated moderate PVL rates exceeding 15% in earlier studies compared with approximately 2.3% for the Sapien 3 [16,17].

### 4.5. Pacemaker Implantation and Conduction Disturbances

A common post-TAVR complication is the need for permanent pacemaker implantation. In our study, pacemaker rate is similar in both groups. Aligning with previous data, the pacemaker rate for the Sapien group was 9.8%. In a meta-analysis, Wang reported a PM rate of 11,5% for the Sapien valve. The 11.8% PPM rate with Navitor is considerably lower than the 21.6% reported by Aoun et al. [8]. Conduction system disturbances occur due to mechanical contact with the membranous septum [19]. Therefore, deep implantation leads to a high risk of pacemaker implantation [20]. Self-expandable valves showed decreasing post-interventional PM rates due to improved implantation techniques, e.g., consistency regarding implantation depth and implantation in cusp overlap projection with a reduced risk of septal trauma [21,22].

### 4.6. Coronary Access Considerations

As TAVR extends to younger and lower-risk populations, maintaining future coronary access has become a pivotal consideration in valve selection.

The intra-annular design and low stent frame of the well-established SAPIEN 3 platform provide unimpeded coronary access, as demonstrated in the ALIGN ACCESS study [23].

The design of the Navitor—with its large open-cell frame and intra-annular position—holds promise for improved post-TAVR coronary access.

This is particularly advantageous over other self-expanding platforms, where commissural misalignment and supra-annular positioning can hinder coronary cannulation. Additionally, low sinus of Valsalva and a higher THV–sinus of Valsalva relation increase the risk of impaired coronary access in patients with a supra-annular THV [23].

The ability to balance optimal hemodynamics with coronary accessibility will be essential in managing patients over a lifetime. Ongoing trials such as the COMFORT study are expected to provide further insights into coronary re-access following Navitor implantation [24].

### 4.7. Stroke and Neurological Safety

Neurological complications remain among the most feared adverse events post-TAVR [25]. There is conflicting data regarding the impact of predilatation as a potential risk factor for stroke [26]. The CENTER2 trial, including 24,305 patients, aimed to assess the incidence, predictors and outcomes of cerebrovascular events following TAVI. While the trial did not specifically address the impact of postdilatation on stroke risk, the association of postdilatation for stroke was evident [27].

In our cohort, these interventions were more frequently required in the Navitor group. Recent head-to-head comparisons have indicated a lower stroke incidence in patients treated with balloon-expandable valves compared to Navitor [27]. However, we observed no statistically significant differences in stroke incidence between groups, suggesting that procedural refinements and operator experience may mitigate these risks. Notably, no disabling strokes occurred in either group during short-term follow-up.

### 4.8. Mortality and Overall Safety

Short-term all-cause mortality was low and did not differ significantly between the Navitor and Sapien groups. Additionally, the rates of serious complications—including life-threatening bleeding, major vascular events, and stroke—were comparable, affirming the safety of the Navitor valve during its early clinical implementation.

When interpreting these results, it is important to consider the chronological context of the device implantations. While the balloon-expandable Sapien platform represents a mature technology with highly standardized deployment techniques refined over nearly a decade at our institution, the Navitor group represents our initial experience with a novel self-expanding design. Remarkably, despite being evaluated during the institutional learning curve of a newly introduced device—when procedural nuances were still being optimized—the Navitor valve demonstrated clear hemodynamic advantages, including significantly lower transvalvular gradients and reduced severe PPM, when compared directly against a highly mature and optimized platform.

## 5. Conclusions

In conclusion, at 30-day follow-up, the self-expanding intra-annular Navitor valve demonstrated excellent acute safety and superior early hemodynamic performance, characterized by significantly lower transvalvular gradients and lower rates of severe PPM compared to the balloon-expandable Sapien 3 Ultra. However, whether these acute hemodynamic advantages translate into superior long-term clinical outcomes, improved valve durability, or facilitated lifetime management remains to be determined in long-term follow-up studies.

## 6. Limitations

First, the major limitation of this study is its short-term follow-up duration, which was strictly limited to 30 days post-procedure. Consequently, while our data provide valuable insights into the acute procedural safety and early hemodynamic profile of the Navitor valve, they cannot support definitive conclusions regarding long-term clinical advantages, structural valve degeneration, or true lifetime management implications. Long-term longitudinal data are needed to confirm these early trends.

Second, due to the retrospective design of this study, detailed systemic flow parameters such as stroke volume and indexed stroke volume were not systematically recorded and were unavailable for the majority of the study population. Consequently, potential confounding by low-flow states on the calculated effective orifice area and patient–prosthesis mismatch cannot be entirely excluded.

Third, the long enrollment window (2015–2023) introduces a potential risk of temporal bias, as it encompasses evolving operator experience and refinements in TAVI techniques. Although the propensity-score-matching model included key clinical risk scores (EuroSCORE II) and precise anatomical metrics to ensure baseline cohort comparability, variations in procedural strategies over time cannot be completely adjusted for. Additionally, although key clinical and anatomical surrogates were rigorously matched, the lack of explicit, independent matching variables for direct quantitative CT calcium scoring, objective frailty scales, and baseline left ventricular ejection fraction remains a limitation inherent to the retrospective nature of this registry data. Furthermore, because the assignment to either the Navitor or Sapien 3 Ultra group was non-randomized and based on operator preference, residual selection bias cannot be completely ruled out, despite the rigorous propensity score matching utilized to balance baseline clinical and anatomical covariates.

## Figures and Tables

**Table 1:** Baseline characteristics before and after matching.

	Prematch				Postmatch			
Variable	Navitor	Sapien	SMD	*p*	Navitor	Sapien	SMD	*p*
	n = 51	n = 1141			n = 51	n = 102		
female gender	26 (51.0%)	424 (37.2%)	0.285	0.055	26 (51.0%)	52 (51.0%)	0	>0.99
age (y)	80.4 ± 6.7	80.8 ± 6.4	0.068	0.7	80.4 ± 6.7	79.9 ± 6.5	0.07	0.7
euroscore 2 (%)	3.4 ± 3.1	4.3 ± 4.9	0.185	0.056	3.4 ± 3.1	3.2 ± 2.7	0.091	0.6
BMI	27.2 ± 4.5	27.3 ± 5.1	0.024	0.9	27.2 ± 4.5	27.9 ± 6.1	0.122	0.4
STS-PROM (%)	3.6 ± 3.4	3.7 ± 3.3	0.013	0.9	3.6 ± 3.4	3.2 ± 2.1	0.173	0.4
EF > 50%	40 (78%)	742 (65%)	0.282	0.051	40 (78%)	82 (80%)	0.049	0.8
EF 35–50%	8 (16%)	271 (24%)	0.19	0.2	8 (16%)	12 (12%)	0.116	0.6
EF < 35%	3 (6%)	128 (11%)	0.171	0.4	3 (6%)	8 (8%)	0.076	0.8
previous pacemaker (n; %)	2 (3.9%)	134 (11.7%)	0.246	0.11	2 (3.9%)	3 (2.9%)	0.055	>0.99
stroke (n; %)	6 (11.8%)	142 (12.4%)	0.021	>0.99	6 (11.8%)	13 (12.7%)	0.03	>0.99
CAD (n; %)	32 (62.7%)	706 (61.9%)	0.018	>0.99	32 (62.7%)	60 (58.8%)	0.08	0.7
annulus area (mm)	447.9 ± 51.1	473.6 ± 188.9	0.139	0.005	447.9 ± 51.1	448.3 ± 50.5	0.008	>0.99
preproc. mean gradient (mmHG)	40 ± 14.5	39 ± 15.6	0.062	0.6	40 ± 14.5	41.9 ± 14.8	0.131	0.4
preproc. max gradient (n; %)	59.7 ± 26.3	62.5 ± 27	0.104	0.5	59.7 ± 26.3	66.5 ± 30.7	0.232	0.2
Perimeter (cm)	7.6 ± 0.4	7.8 ± 0.7	0.213	0.022	7.6 ± 0.4	7.6 ± 0.4	0.01	>0.99
Diameter (mm)	24.1 ± 1.4	26.8 ± 28	0.097	0.002	24.1 ± 1.4	24.1 ± 1.4	0.008	>0.99

Groups were compared using fisher-exact-test or t-test. SMD: Standardized mean difference, EF = Ejection-Fraction.

**Table 2:** Procedural details.

Variable	All (n = 153)	Navitor (n = 51)	Sapien (n = 102)	*p*-Value
**Implanted Valve Size:**				
Navitor 25	10 (6.5%)	10 (19.6%)	0 (0.0%)	
Navitor 27	23 (15.0%)	23 (45.1%)	0 (0.0%)	
Navitor 29	18 (11.8%)	18 (35.3%)	0 (0.0%)	
Sapien 23	29 (19.0%)	0 (0.0%)	29 (28.4%)	
Sapien 26	71 (46.4%)	0 (0.0%)	71 (69.6%)	
Sapien 29	2 (1.3%)	0 (0.0%)	2 (2.0%)	
Procedural time (min)	58 [17–270]	63 [39–155]	54 [17–270]	0.002
Contrast (ml)	115 [50–430]	150 [84–430]	110 [50–330]	<0.001
Fluoroscopy time (min)	11.1 [2.8–35.4]	12.2 [5.9–31.8]	10.3 [2.8–35.4]	0.011
Predilatation	68 (44.4%)	50 (98.0%)	18 (17.6%)	<0.001
Postdilatation	52 (34.0%)	23 (45.1%)	29 (28.4%)	0.047

**Table 3:** In hospital outcomes.

Variable	All (n = 153)	Sapien (n = 102)	Navitor (n = 51)	*p*-Value
30 days mortality	1 (0.7%)	0 (0.0%)	1 (2.0%)	0.3
New pacemaker	16 (10.5%)	10 (9.8%)	6 (11.8%)	0.8
Stroke	1 (0.7%)	1 (1.0%)	0 (0.0%)	0.1
Life threatening bleeding	5 (3.3%)	3 (2.9%)	2 (3.9%)	>0.99
Major bleeding	3 (2.0%)	3 (2.9%)	0 (0.0%)	0.55
Major vascular complications	4 (2.6%)	3 (2.9%)	1 (2.0%)	>0.99
Minor vascular complications	23 (15.0%)	14 (13.7%)	9 (17.6%)	0.6

**Table 4:** VARC-3 defined composite outcomes.

Variable	All (n = 153)	Sapien (n = 102)	Navitor (n = 51)	*p*-Value
Technical success	143 (93.5%)	95 (93.1%)	48 (94.1%)	>0.99
Device success	130 (85.0%)	83 (81.4%)	47 (92.2%)	0.8

## Data Availability

The datasets generated and/or analyzed during the current study are not publicly available due to patient privacy restrictions but are pseudonymized and available from the corresponding author upon reasonable request.

## Abbreviations

TAVI	Transcatheter Aortic Valve Implantation
TAVR	Transcatheter Aortic Valve Replacement
THV	Transcatheter Heart Valve
NAV/Navitor	Abbott Navitor Transcatheter Heart Valve
S3/S3U	Edwards Sapien 3/Sapien 3 Ultra Valve
SAVR	Surgical Aortic Valve Replacement
CAD	Coronary Artery Disease
EOA	Effective Orifice Area
PVL	Paravalvular Leak (or Regurgitation)
PPM	Patient–Prosthesis Mismatch
VARC-3	Valve Academic Research Consortium-3
EF	Ejection Fraction
SD	Standard Deviation
SMD	Standardized Mean Difference
PM	Pacemaker
LVEF	Left Ventricular Ejection Fraction
LVOT	Left Ventricular Outflow Tract
